# Shaping the Future of Digital Health Education in Canada: Prioritizing Competencies for Health Care Professionals Using the Quintuple Aim

**DOI:** 10.2196/75904

**Published:** 2025-09-08

**Authors:** Glynda Rees, Lorelli Nowell, Tracie Risling

**Affiliations:** 1 School of Health Sciences, British Columbia Institute of Technology, 3700 Willingdon Avenue, Burnaby, BC, V5G 3H2, Canada, 1 604-765-8913; 2 Doctoral Student, Faculty of Nursing, University of Calgary, Calgary, AB, Canada; 3 Faculty of Nursing, University of Calgary, Calgary, AB, Canada

**Keywords:** digital health, health informatics, competency-based education, Quintuple Aim

## Abstract

The integration of digital health and informatics competencies into health care education in Canada is essential for preparing a workforce capable of leveraging health care technologies to enhance care delivery and patient outcomes. Despite significant advancements, the current educational landscape in digital health remains inconsistent, characterized by fragmented curricula and uneven competency attainment. Addressing these gaps requires an innovative reframing of digital health competencies guided by a robust, outcomes-oriented framework. These authors propose the Quintuple Aim as an effective framework for outlining and organizing digital health and informatics competencies, focusing simultaneously on improving patient experience, enhancing population health, reducing health care costs, improving health care provider experience, and advancing health equity. Each dimension of the Quintuple Aim provides a critical lens for identifying, prioritizing, and contextualizing core competencies. Within the “patient experience” aim, competencies prioritize patient-centered technology use, including digital literacy, privacy awareness, and the ability to empower patients through technology. “Healthcare provider experience” competencies prioritize usability, workflow integration, and strategies to mitigate technology-related burnout. Under “population health,” competencies emphasize data-driven decision-making, analytics, and health informatics to support effective public health interventions. Competencies associated with “cost reduction” focus on operational efficiency, resource optimization, and economic evaluation of digital health solutions. Finally, “health equity” competencies emphasize inclusivity, cultural safety, and the elimination of digital divides, ensuring equitable access to digital health technologies. Potential assessment strategies aligned with each competency area are highlighted, emphasizing formative and summative evaluations that include simulation-based assessments, real-world technology integration projects, and reflective practice portfolios. By applying the Quintuple Aim as a guiding structure, digital health education can achieve greater standardization, clarity, and alignment with health care system needs, while simultaneously allowing for tailored adaptations responsive to specific regional and institutional priorities. This paper introduces the Quintuple Aim as a guiding framework to comprehensively identify and organize core digital health and informatics competencies for health professional education.

## Introduction

The rapid advancement of digital health technologies necessitates a health care workforce equipped with the requisite knowledge, skills, and abilities to effectively integrate and use these technologies [1-3]. Educating the Canadian health care workforce in digital health is essential to enhance patient care and safety, improve health care accessibility, and ensure the sustainability of health care systems. The International Council of Nurses, the World Health Organization (WHO), and the Organisation for Economic Co-operation and Development (OECD) emphasize the importance of digital literacy for health care providers to effectively leverage technology and adapt to evolving health care challenges [4-7]. Despite the increasing importance of digital health and health informatics, there is a significant gap in the number of educational programs offered across Canada that prepare health care professionals for these challenges [2,8].

There is a recognized and acute need for more digital health professionals who bring informatics knowledge, digital health understanding, and data analytics skills to health care settings [2,5,8-12]. Terms essential to this discussion include digital health, health informatics, and competency. Digital Health refers to the use of technologies for improving the health and well-being of people at individual and population levels, as well as enhancing the care of patients through intelligent processing of clinical and genetic data and leveraging digital health technologies and services to transform care delivery [5,7,13,14]. The Canadian Institutes of Health Research cite Canada Health Infoway’s definition of Digital Health as “the use of information technology or electronic communication tools, services and processes to deliver health care services or to facilitate better health” [15]. Health Informatics is a focused area within digital health and is defined by the American Medical Informatics Association as “the science of how to use data, information and knowledge to improve human health and the delivery of health care services” [16]. A competency refers to defined knowledge, skills, and qualities expected to competently carry out a function [17]. In the context of health care, the term competency is defined as “an observable ability of a health professional, integrating multiple components such as knowledge, skills and attitudes” [18]. Informatics competencies are integral to the broader concept of digital health and should be integrated into an educational digital health program [1,2,8]. For the purposes of this paper, digital health is used as an umbrella term that includes health informatics competencies alongside broader digital technologies and services that enhance health system performance. These competencies are designed for health care professionals seeking digital health education to develop the knowledge and skills necessary for effectively integrating digital technologies into health care practice.

## Current State of Digital Health Education

Despite multiple organizations highlighting the need for the health care workforce to acquire and maintain the knowledge and skills to navigate technologically advancing health care systems [2,5,9-12], current Canadian postsecondary educational programs often lack the comprehensive curriculum, faculty expertise, and resources necessary to equip graduates with these competencies [2,8]. This gap between the evolving digital health landscape and the preparedness of health care professionals can potentially lead to inefficiencies, reduced quality of care, increased clinician workload, and missed opportunities for innovation within health care settings [9,10].

If the gap in digital health education remains unresolved, the health care workforce will continue to struggle with the integration and use of digital health technologies, which are increasingly vital for modern health care delivery. The International Council of Nurses highlights that digital health is integral to enhancing care quality and patient outcomes, making it essential for nurses to be proficient in these technologies [5]. The WHO also stresses that a well-prepared workforce is crucial to achieving the full potential of digital health innovations globally [7]. Without adequate education, the health care sector risks failing to capitalize on digital evolutions as noted by Socha-Dietrich [6], whose OECD report emphasizes the importance of empowering health care professionals to adapt to these advancements. Furthermore, the 2023 Canadian Survey of Nurses by Canada Health Infoway reveals that gaps in digital health proficiency can directly impact the quality of care provided [19].

## Prioritizing Digital Health and Informatics Competencies

Digital health technologies are transforming health care delivery, requiring a shift in education to equip health care professionals with essential digital health and informatics competencies. Effective digital health educational programs typically include components such as data analytics, health information system interoperability, privacy and ethical considerations, and health informatics theory [20]. These curricula also emphasize skills such as leadership, communication, project management, and change management, all of which are critical in implementing and managing digital health initiatives [21]. Studies have shown that graduates of digital health programs often demonstrate improved competencies in using digital health technologies, which translates to better patient outcomes and more efficient health care delivery [10,20-22].

## The Quintuple Aim as a Framework

The Quintuple Aim is a framework for health care improvement that aims to make health care more equitable, effective, and efficient [23]. In the evolving landscape of health care, the integration of digital health and informatics competencies plays a critical role in achieving the goals outlined in the Quintuple Aim: enhancing patient experience, improving care team well-being, reducing costs, advancing population health, and promoting health equity [23,24]. These competencies empower providers to harness technology and data-driven insights, ultimately enhancing patient care and health outcomes. By aligning digital health and informatics competencies with the Quintuple Aim, health care professionals can better navigate and contribute to a system that prioritizes both efficiency and equity.

Many researchers and organizations have compiled lists of informatics competencies for health care professionals in various roles in the health care system [20-22,25-29,30,31]. In addition, several researchers have developed frameworks to guide the organization, prioritization, and context of the competencies [21,22,32-36]. These authors suggest organizing and combining these competencies into the 5 categories outlined in the Quintuple Aim (patient experience, provider experience, reduced cost, population health, and health equity). This competency framework supports clinicians, health care administrators, informatics specialists, and other professionals preparing for roles in digital health. These competencies are not only confined to single objectives but intersect across multiple areas of the Quintuple Aim. As health care systems increasingly adopt advanced digital solutions, it becomes evident that these competencies do not function in isolation; rather, they create synergies that benefit both individual and population-level outcomes. Understanding these overlaps is essential for designing education programs and initiatives that prepare health care providers to meet the complex and interconnected needs of today’s health care environments. Table 1 maps each of the Quintuple Aims to digital health competencies as well as potential assessment strategies.

**Table 1.** Mapping digital health competencies to the Quintuple Aim Framework.

Quintuple Aim and competency	Assessment strategies
Patient experience	
Integrating digital health technologies into care	Practical simulations, case studies, and reflective assignments around patient management, communication, and decision-making, ethical, privacy, and interoperability concerns.
Applying and understanding user-centered design	Project-based evaluations to design, test, and refine digital health solutions based on patient needs, usability, and accessibility.
Patient-centered digital literacy and virtual care skills	Role play scenarios and patient simulations to communicate via digital platform and ensure patients can navigate virtual care services.
Ethical practice	Case-based discussions, ethical dilemma scenarios, and reflective essays around privacy, data security, informed consent, and responsible use of technology.
Communication skills	Virtual patient consultations and collaborative team simulations to explain digital health tools, address patient concerns, and facilitate effective communication through digital platforms.
Genomics competencies	Case studies, quizzes, or practical exercises to interpret genetic data, apply genomic information in clinical decision-making, and use digital tools for personalized patient care.
Provider experience	
Interoperability principles	Scenario-based assignments and systems integration projects to navigate and connect various digital health platforms, ensuring seamless data exchange and collaboration across health care settings.
User-centered design and workflow optimization	Hands-on projects to design digital health solutions that prioritize user experience, streamline clinical processes, and demonstrate the ability to enhance efficiency and usability within health care environments.
Critical thinking and evaluation	Analytical case studies to critically assess the effectiveness of digital health tools, identify potential areas for improvement, and evaluate their impact on patient care and health care workflows.
Clinical decision support and evidence-based practice	Simulations and practical examinations to use digital tools to make data-driven decisions, incorporate the latest clinical guidelines, and evaluate the outcomes of their decisions in patient care scenarios.
Reduced cost	
Efficient use of digital health technologies	Real-world simulations and timed exercises to quickly and accurately navigate digital health systems, manage patient data, and complete tasks with minimal errors and maximum efficiency.
Cybersecurity, privacy, and ethical use of digital health technologies	Written examinations, case studies, and scenario-based discussions to test knowledge of data protection laws, ethical guidelines, and strategies for safeguarding patient information in digital health environments.
Digital health ecosystems and interoperability	Group projects to analyze and design solutions that demonstrate how different digital health platforms work together.
Data analysis	Data-driven case studies and analytical projects to interpret health care data, identify trends, and make recommendations.
Population health	
Data management and analysis	Projects to analyze large data sets to identify trends and evaluate strategies.
Adaptability to emerging technologies	Hands-on exercises and simulations to learn and integrate new tools into clinical practice.
Leadership, change management, and project management skills	Team-based projects to plan, execute, and evaluate implementation of digital health solutions.
Health equity	
Addressing digital health disparities	Case studies and community-based projects to identify barriers to digital health access, propose strategies for increasing inclusivity, and evaluate the impact of their solutions on underserved populations.
Health data access	Practical exercises and written assignments to analyze the ethical, legal, and technical aspects of accessing and sharing health data, ensuring compliance with regulations while considering patient privacy and data security.
Indigenous data sovereignty	Case studies and reflective essays that analyze the importance of respecting Indigenous peoples’ control over their health data, explore ethical considerations, and demonstrate how to apply culturally appropriate practices in digital health.
Health literacy and patient education	Role-playing exercises and patient simulation scenarios to effectively communicate digital health information, assess patient comprehension, and tailor education to diverse literacy levels and needs.

## Patient Experience

### Overview

The patient experience category of the Quintuple Aim focuses on improving the quality, accessibility, and personalization of care to enhance patient satisfaction and outcomes. Digital health technologies play a crucial role in advancing this goal by enabling virtual care, mobile health apps, wearable devices, and patient portals that facilitate seamless communication between patients and providers. These tools empower individuals to actively engage in their care, access real-time health information, and receive timely interventions, ultimately leading to more patient-centered, efficient, and responsive health care experiences.

### Integrating Digital Health Technologies Into Care

The ability to effectively translate digital health knowledge and theory into practical applications within patient care settings is essential to enhancing the patient experience with digital health technologies. Health care students should be supported in developing proficiency in using digital health technologies and applications for patient care, identifying appropriate patient education materials, and acting as advocates for system users [2,37]. Competencies in using electronic health records for comprehensive documentation, effective communication among care providers, and making evidence-informed decisions are needed in today’s health care workforce [4,5,28]. Health care students should be adept at leveraging virtual care platforms for remote consultations and patient monitoring, expanding access to care and addressing health care disparities [10,25,26]. In addition, understanding how digital tools can enhance patient engagement and empower individuals to actively participate in their care and self-management of chronic conditions is essential [11]. Understanding the functionalities and interconnectedness of health information systems, virtual care platforms, mobile health apps, and emerging technologies like artificial intelligence (AI) and robotics is fundamental [1,3,38-40]. Furthermore, understanding the capabilities and limitations of these technologies is essential for their appropriate and effective application in providing quality patient care. If digital health technologies are not effectively integrated into patient care, patients may face reduced access to timely information, fragmented communication with providers, and lower engagement in their own health management, ultimately compromising their overall health care experience. The competency of integrating digital health technologies into care could be assessed through practical simulations, case studies, and reflective assignments that evaluate health care students’ competency in using digital tools for patient management, communication, and decision-making, while also considering their understanding of ethical, privacy, and interoperability concerns (Table 1).

### Applying and Understanding User-Centered Design

Informatics competencies that enhance patient-centered care focus on human-centered design, usability principles, and patient engagement strategies within digital health solutions. Students should learn to critique digital health technologies, such as patient portals, telehealth and virtual care systems, and mobile health apps, to ensure they are user-friendly, accessible, and culturally sensitive [41,42]. Training in health literacy principles and user-centered design methodologies can foster empathy by deepening health care students' understanding of patient needs, ensuring that digital health solutions are accessible, inclusive, and tailored to diverse populations. If health care professionals understand and apply user-centered design, it will make it easier for patients to navigate and engage with health technologies, ultimately leading to improved patient satisfaction [43]. If user-centered design is not applied when integrating digital health technologies, patients may struggle with inaccessible, confusing, or nonintuitive systems, leading to frustration, decreased engagement, and potential disparities in health care access and outcomes. Health care students’ abilities to apply and understand user-centered design in relation to digital health technologies could be assessed through project-based evaluations where students design, test, and refine digital health solutions, considering patient needs, usability, and accessibility to ensure the technology meets the diverse requirements of end users (Table 1).

### Patient-Centered Digital Literacy and Virtual Care Skills

For patients and caregivers, digital health literacy is crucial for understanding and managing their own health conditions. With the advent of virtual care, online patient portals, and mobile health apps, patients are increasingly expected to engage with digital platforms [44]. The increased adoption of virtual care, accelerated by the COVID-19 pandemic, underscores the need for health care professionals to be proficient in patient-centered digital literacy [45,46]. This includes using virtual care platforms and mobile health apps effectively to engage with patients remotely [47]. Health care professionals are often required to support patients in using digital health technologies to manage their health [2,48]. Patient-centered digital literacy and virtual care competencies are particularly important as virtual care has become a staple in health care delivery, requiring providers to ensure equitable access and appropriate care via digital means [42,45,46,49]. A lack of digital health literacy can lead to misunderstandings, nonadherence to treatment plans, and ultimately poorer health outcomes [50]. Competencies in patient-centered digital literacy and virtual care skills could be assessed through role-playing scenarios, patient simulations, and assessments that measure health care students’ ability to effectively communicate with patients via digital platforms, educate them about digital tools, and ensure patients can navigate virtual care services with confidence and ease (Table 1).

### Ethical Practice

Ethical training on the appropriate use of digital tools is necessary to navigate complex privacy issues and ensuring health care professionals are equipped with these skills fosters secure and ethical practices in digital health environments [27,51]. In addition, competencies in data privacy, confidentiality, and security are essential, as safeguarding patient data contributes to clinicians’ confidence and comfort in using digital health systems, thereby supporting a safer work environment [52]. Furthermore, integrating ethical frameworks into digital health education can help health care professionals recognize and address biases, promote equitable access to technology, and uphold patient autonomy in an increasingly digital health care landscape. If ethical practices are not incorporated into digital health education, there is a risk of compromising patient privacy, consent, and autonomy, which can undermine trust in health care systems and negatively impact patient experiences [52]. Ethical practice competencies may be assessed through case-based discussions, ethical dilemma scenarios, and reflective essays that evaluate health care students' understanding of privacy, data security, informed consent, and the responsible use of technology in patient care (Table 1).

### Communication Skills

Strong communication skills are critical for the effective use of digital health technologies to interact with patients, explain complex information, and provide clear instructions [51]. For instance, explaining the use of health information technologies, providing educational resources on conditions, treatments, procedures, or guiding patients on using digital health apps requires excellent communication skills. Health care professionals must also be trained in communication competencies, such as digital compassion and patient advocacy, so as to better facilitate positive experiences for patients [20,21,53,54]. These competencies are particularly crucial in virtual care, where fostering patient trust and engagement without face-to-face interaction demands a specialized skill set [45,46]. If communication skills are not incorporated into digital health education, patients may experience a lack of emotional connection with health care providers, leading to diminished patient satisfaction and outcomes. Communication skills may be assessed through interactive exercises, such as virtual patient consultations or collaborative team simulations, where health care students are evaluated on their ability to clearly explain digital health tools, address patient concerns, and facilitate effective communication through digital platforms (Table 1).

### Genomics Competencies

Genomic competencies empower health care providers to deliver more precise and personalized care, identifying genetic factors that influence disease risk, medication responses, and treatment options [41]. By integrating genomic data into clinical decision-making, practitioners can tailor interventions to each patient’s unique genetic profile, potentially improving the efficacy of treatments and reducing adverse reactions to medications [41,55,56]. As genomic technology continues to evolve, training in this area not only supports patient outcomes but also contributes to more efficient, cost-effective, and equitable health care by reducing trial-and-error in treatments and focusing on individualized approaches [55-58]. Without adequate training in genomics, health care professionals may struggle to interpret complex genetic data, limiting their ability to leverage these advancements in clinical practice and potentially widening disparities in access to precision medicine. Genomics competencies may be assessed through case studies, quizzes, and practical exercises that test health care students’ ability to interpret genetic data, apply genomic information in clinical decision-making, and use digital tools for personalized patient care (Table 1).

## Provider Experience

### Overview

The provider experience category of the Quintuple Aim emphasizes the well-being, satisfaction, and efficiency of health care professionals, recognizing that a supported workforce is essential for high-quality patient care. Digital health technologies contribute to this goal by streamlining workflows, reducing administrative burdens, and enhancing communication through electronic health records, clinical decision support systems, and telehealth platforms. These tools can help mitigate burnout by improving efficiency, reducing repetitive tasks, and allowing providers to focus more on patient care. When effectively integrated, digital health solutions potentially foster a more sustainable and rewarding work environment for health care professionals.

### Interoperability Principles

Understanding interoperability standards to facilitate enhanced care coordination can help leverage the full potential of digital health technologies [9]. This encompasses an understanding of interoperability and data standards, enabling the seamless exchange of health information between different systems and care settings [59]. As scopes of practice shift and we are faced with workforce shortages and the emergence of new roles, having access to comparable, sharable data derived from the use of clinical data standards has the potential to provide support for human resource and health policy decisions at local, regional, and national levels [60,61]. As we plan for a future where AI is foundational to facilitate evidence-informed decision-making, it is vital that we have standardized clinical data to inform care decisions and resource allocation. If interoperability principles are not incorporated into digital health education, health care providers may face fragmented systems, difficulty accessing patient information across platforms, and increased administrative burden, all of which can hinder clinical decision-making and job satisfaction [52]. Health care students’ understanding and use of interoperability principles could be assessed through scenario-based assignments and system integration projects where they demonstrate their ability to navigate and connect various digital health platforms, ensuring seamless data exchange and collaboration across health care settings (Table 1).

### User-Centered Design and Workflow Optimization

Designing user-friendly digital health systems that minimize cognitive load and enhance workflow efficiency can significantly improve provider well-being [34]. Digital health education programs should include principles of human-computer interaction, usability testing, and ergonomics to ensure systems are intuitive and supportive of provider workflows [4,5,28,34]. Digital health systems generate numerous alerts and notifications, potentially contributing to provider burnout. Training can address strategies for managing alert fatigue, including customization, prioritization, and effective communication protocols to minimize unnecessary interruptions and streamline information flow [62].

Key competencies also include ergonomics, cognitive load theory, and human factors in technology design. These informatics skills empower health care providers to design and implement systems that minimize administrative burden, optimize usability, and reduce unnecessary interruptions during clinical workflows [63]. For example, well-designed electronic health record interfaces that prioritize intuitive navigation and minimize excessive documentation can significantly reduce cognitive overload, allowing providers to focus more on direct patient care.

Training in workflow optimization and data automation is also vital, as these competencies enable professionals to implement time-saving measures within electronic health record systems and other digital health tools. Reducing the time spent on repetitive or low-value tasks allows clinicians to dedicate more time to patient care, which can enhance job satisfaction and reduce burnout [63]. In addition, an understanding of implementation science, which guides the successful integration of new technologies into health care systems, may help optimize workflow by identifying barriers to technology adoption, refining processes to enhance efficiency, and ensuring digital tools seamlessly integrate into clinical practice [10]. If user-centered design and workflow optimization are not incorporated into digital health education, health care providers may encounter inefficient, cumbersome systems that increase cognitive load, disrupt clinical workflows, and contribute to burnout and frustration. User-centered design and workflow optimization could be assessed through hands-on projects where health care students design digital health solutions that prioritize user experience, streamline clinical processes, and demonstrate the ability to enhance efficiency and usability within health care environments (Table 1).

### Critical Thinking and Evaluation

The rapid evolution of the digital health landscape necessitates the ability to critically evaluate new tools, discern their potential benefits and limitations, and assess their impact on health care delivery and outcomes [48,64]. This competency involves building a strong theoretical understanding of health informatics, staying abreast of emerging technologies, recognizing their strengths and weaknesses, and developing the ability to translate data and research findings into evidence-informed practices. A strong understanding of data science and bioinformatics can further enhance the ability to critically analyze health data and contribute to the development of novel digital health solutions [42]. If critical thinking and evaluation are not incorporated into digital health education, health care providers may struggle to assess the effectiveness of digital tools, leading to suboptimal decision-making, reduced confidence in technology, and potential negative impacts on patient care. Critical thinking and evaluation skills could be assessed through analytical case studies, where health care students critically assess the effectiveness of digital health tools, identify potential areas for improvement, and evaluate their impact on patient care and health care workflows (Table 1).

### Clinical Decision Support and Evidence-Based Practice

Digital technologies that enhance evidence-informed practice, such as clinical decision support systems, are becoming central to health care delivery. Educating clinicians on the use and optimization of these tools helps improve diagnostic accuracy, decision-making, and treatment planning [20]. Furthermore, familiarity with decision support technologies enables health care professionals to access real-time evidence, which is crucial for safe, high-quality care [20]. Failing to advance the use of clinical decision support and evidence-based practice using digital technologies may lead to outdated treatments, increased medical errors, and missed opportunities for improving patient outcomes through data-driven insights. Clinical decision support and evidence-based practice skills may be assessed through simulations or practical examinations where health care students use digital tools to make data-driven decisions, incorporate the latest clinical guidelines, and evaluate the outcomes of their decisions in patient care scenarios (Table 1).

## Reduced Cost

### Overview

The reduced costs category of the Quintuple Aim focuses on lowering health care expenses while maintaining or improving the quality of care. Digital health technologies contribute significantly to cost reduction by streamlining administrative tasks, reducing hospital readmissions, and enabling remote monitoring through telehealth and mHealth apps. By automating routine processes, improving care coordination, and providing patients with more proactive management of chronic conditions, these technologies can minimize the need for costly in-person visits and prevent unnecessary hospitalizations. Ultimately, the integration of digital tools could create a more efficient and financially sustainable health care system.

### Efficient Use of Digital Health Technologies

Proficiency in using electronic health records to streamline documentation, reduce redundancy, and improve care coordination can lead to significant cost savings. Training should go beyond basic data entry and focus on leveraging decision support systems, expert systems, and other features to optimize clinical workflows and resource usage [37,48]. Telehealth adoption is rapidly increasing, offering cost-effective virtual care delivery models. Digital health education programs should incorporate telehealth and virtual care training, equipping providers with the skills to conduct virtual visits, remotely monitor patients, and use virtual care platforms effectively [2,45-47]. To help empower informatics professionals to advocate for cost-effective technology adoption and usage, they should also be aware of the economic implications of digital health technologies, including cost-benefit analysis, return on investment, and value-based health care models [34]. If the efficient use of digital health technologies is not incorporated into digital health education, health care systems may face increased operational costs due to inefficiencies, higher rates of preventable hospitalizations, and missed opportunities for cost-saving innovations such as telehealth and automation. Efficient use of digital health technologies could be assessed through timed exercises or real-world simulations where health care students demonstrate their ability to quickly and accurately navigate digital health systems, manage patient data, and complete tasks with minimal errors and maximum efficiency (Table 1).

### Cybersecurity, Privacy, and Ethical Use of Digital Health Technologies

With the growing reliance on digital tools comes the increased risk of data breaches and cyberattacks. Competency in cybersecurity, including understanding data protection principles and mitigating security risks, is essential for maintaining patient trust and ensuring the safety of health information systems [65]. Cybersecurity, privacy, and ethical use of digital health technologies reduce health care costs by safeguarding sensitive information, preventing data breaches, and ensuring trust in digital systems. Effective cybersecurity measures prevent costly breaches that can lead to financial penalties, legal liabilities, and expensive recovery processes [65]. In addition, ethical use of digital health technologies ensures transparency, fostering patient trust, which encourages technology adoption and self-management [27]. If cybersecurity, privacy, and the ethical use of digital health technologies are not incorporated into digital health education, health care systems may face costly data breaches, legal penalties, and loss of patient trust, ultimately leading to increased financial and reputational risks. Cybersecurity, privacy, and the ethical use of digital health technologies may be assessed through written examinations, case studies, or scenario-based discussions that test health care students’ knowledge of data protection laws, ethical guidelines, and strategies for safeguarding patient information in digital health environments (Table 1).

### Digital Health Ecosystems and Interoperability

Promoting interoperability and connected care in health care reduces costs by improving coordination and access to complete patient information, which decreases unnecessary tests, procedures, and medication errors [52]. These systems streamline administrative tasks, lowering overhead, and support value-based care models focused on quality outcomes. In addition, by enabling population health management and enhancing patient engagement, interoperability fosters preventive care and self-management, further lowering expenditures associated with chronic conditions and emergency care. Overall, interoperability creates a more efficient, cohesive, and cost-effective health care environment [52,66]. If digital health ecosystems and interoperability are not incorporated into digital health education, health care organizations may incur higher costs due to redundant testing, inefficiencies in data sharing, and fragmented care coordination, leading to suboptimal resource use. Health care students’ understanding of digital health ecosystems and interoperability could be assessed through group projects where they analyze and design solutions that demonstrate how different digital health platforms can communicate and work together to improve patient care and health care outcomes (Table 1).

### Data Analysis

The reduction of health care costs can be achieved through the application of informatics competencies such as data analysis. Cost analysis skills help professionals make data-informed decisions that balance cost and quality in patient care, while health economics education emphasizes understanding economic drivers within health care [34]. Cost-benefit analysis tools within digital health platforms allow for optimized resource allocation and reduced waste, ultimately reducing costs while maintaining quality. Through these competencies, digital health practitioners can advocate for financial strategies that lower costs without compromising care quality. If data analysis is not incorporated into digital health education, health care systems may struggle to identify cost-saving opportunities, leading to inefficient resource allocation, increased waste, and missed chances for data-driven decision-making that could enhance financial sustainability. The data analysis competency may be assessed through data-driven case studies or analytical projects where health care students interpret health care data, identify trends, and make evidence-based recommendations for improving patient outcomes or optimizing health care processes (Table 1).

## Population Health

### Overview

The population health category of the Quintuple Aim focuses on improving health outcomes across communities by addressing preventive care, chronic disease management, and health disparities. Digital health technologies support this goal by enabling large-scale data collection, predictive analytics, and remote monitoring, allowing health care providers to identify trends, allocate resources efficiently, and implement targeted interventions. Tools such as electronic health records, virtual care platforms, and mobile health apps enhance access to care, especially for underserved populations, while data-driven insights help inform public health strategies. By leveraging digital health, health care systems can promote proactive, equitable, and efficient approaches to population health management.

### Data Management and Analysis

To improve population health, health care professionals must be skilled in using data analytics to assess and address health trends and disparities. Health informatics education programs should thus prioritize competencies in population health informatics, which includes training in data collection, analysis, and interpretation related to population health metrics [20,21]. In addition, familiarity with tools for epidemiological analysis and population health management, including geospatial mapping and predictive analytics, can equip graduates with the knowledge to identify at-risk populations and forecast public health trends [21,25].

Understanding social determinants of health and leveraging big data are also central to enhancing population health, as they enable health care providers to integrate nonclinical factors impacting patient outcomes into their care approaches [42,49]. Analysis of social, behavioral, and environmental determinants of health supports the delivery of personalized care. The ability to collect, store, manage, analyze, and interpret health data is indispensable in the digital health era.

This competency also encompasses data literacy and the ability to critically appraise data quality and reliability [59]. Students should develop proficiency in basic statistics, data visualization techniques, and gain an understanding of data governance and privacy principles, which are particularly critical given the sensitive nature of health information and the need to maintain patient confidentiality [28,34]. As digital health technologies become increasingly sophisticated in capturing social, behavioral, and environmental determinants of health, health care professionals must be able to analyze and use this data to deliver personalized and holistic care, as well as appreciate the extent that digital health technologies impact the planet [2,67-69]. If data management and analysis are not incorporated into digital health education, health care systems may struggle to track disease patterns, allocate resources effectively, and develop targeted public health interventions, ultimately hindering efforts to improve population health outcomes. Health care students’ understanding of data management and analysis in relation to digital health and population health could be assessed through projects where they analyze large datasets to identify health trends, evaluate interventions, and propose strategies for improving population health outcomes using digital tools and evidence-based insights (Table 1).

### Adaptability to Emerging Technologies

AI is being increasingly used to enhance population health strategies through predictive analytics. Health care leaders must stay abreast of emerging technologies such as AI, robotics, and big data analytics [21,40,70]. AI marks a pivotal advancement in the digital transformation of health care, and traditional health care education and training often do not sufficiently address the digital competencies required for its effective use [40]. To ensure the safe and efficient adoption of AI, health care professionals need foundational knowledge in machine learning and neural networks, skills for critical evaluation of datasets, and the ability to integrate AI into clinical workflows while managing bias and ensuring smooth human-machine interaction in clinical settings. Furthermore, an understanding of the legal and ethical implications of digital health and the broader impact of AI adoption is essential [3,12,33,39,49,70]. If adaptability to emerging technologies is not incorporated into digital health education, health care systems may fall behind in implementing innovative solutions, limiting their ability to address evolving public health challenges and widening disparities in access to effective care. Adaptability to emerging technologies may be assessed through hands-on exercises or simulations where health care students are tasked with quickly learning and integrating new digital health tools into clinical practice, demonstrating flexibility and problem-solving skills in response to evolving health care technologies (Table 1).

### Leadership, Change Management, and Project Management Skills

Health care professionals should be equipped to champion digital health transformation and spearhead initiatives that promote the adoption and integration of digital technologies within health care organizations and systems [27,42,59]. This competency includes a grasp of change management principles, enabling graduates to navigate the complexities and challenges associated with implementing new technologies and workflows [20,21]. Proficiency in project management is also vital, ensuring the organized planning, execution, and evaluation of digital health initiatives [20,21]. If leadership, change management, and project management skills are not incorporated into digital health education, health care organizations may struggle to implement and scale digital health initiatives effectively, leading to fragmented care, inefficient resource use, and missed opportunities to improve health outcomes. Leadership, change management, and project management skills in relation to digital health could be assessed through team-based projects where health care students plan, execute, and evaluate the implementation of digital health solutions, demonstrating their ability to lead, manage stakeholder expectations, and navigate challenges in health care technology integration (Table 1).

## Health Equity

### Overview

The health equity category of the Quintuple Aim focuses on reducing disparities in health care access, quality, and outcomes across diverse populations. Digital health technologies support this goal by expanding access to care through telemedicine, mobile health apps, and remote monitoring, particularly for underserved and rural communities. In addition, data analytics and AI can help identify health disparities, enabling targeted interventions and resource allocation. However, to fully realize these benefits, digital health solutions must be designed with inclusivity in mind, ensuring accessibility for individuals with varying levels of digital literacy, socioeconomic backgrounds, and health care needs.

### Addressing Digital Health Disparities

Digital health interventions must be designed to avoid widening existing health inequities. Training should highlight the role of social determinants and the need for equitable technology use, as well as the role that data standards play in ensuring clinical documentation accurately reflects representation of social determinants of health [58]. Culturally sensitive and accessible digital tools are essential, and education programs must stress the importance of cultural considerations to ensure fair access [42,45,49]. Health care professionals also need advocacy skills for the ethical deployment of digital health solutions to prevent these technologies from exacerbating disparities [37]. If addressing digital health disparities is not incorporated into digital health education, existing health care inequities may worsen, as underserved populations could face barriers to accessing digital tools, leading to gaps in care and poorer health outcomes. Health care students’ ability to address digital health disparities may be assessed through case studies or community-based projects where they identify barriers to digital health access, propose strategies for increasing inclusivity, and evaluate the impact of their solutions on underserved populations (Table 1).

### Health Data Access

Limited access to personal health data leads to fragmented care, delays in diagnosis, and increased health care costs [52,66]. Ensuring patients have control over their health data is vital for improving outcomes, especially for marginalized communities [42,71]. Limited access disproportionately affects vulnerable groups, reinforcing health disparities and increasing the economic burden on both individuals and the health care system. Equitable access to health data is crucial for high-quality care and patient autonomy [42,66]. Importantly, “health data access” encompasses both individual-level access, where patients control, view, and share their personal health records, and population-level access, where aggregated data systems support public health surveillance, population health planning, and policy development. Competent digital health professionals must understand and ethically navigate both dimensions to effectively address inequities and improve community health outcomes [72]. If health data access is not incorporated into digital health education, marginalized communities may face continued barriers to care, as health care providers could lack the tools and knowledge to leverage data effectively, exacerbating disparities in health outcomes and access to services. Understanding of health data access could be assessed through practical exercises or written assignments where health care students analyze the ethical, legal, and technical aspects of accessing and sharing health data, ensuring compliance with regulations while considering patient privacy and data security (Table 1).

### Indigenous Data Sovereignty

Understanding Indigenous Data Sovereignty helps professionals create data systems respectful of Indigenous cultural and ethical values. Principles like OCAP (Ownership, Control, Access, Possession) emphasize self-determination, advocating for ethical guidelines that honor Indigenous perspectives on health data [73,74]. Indigenous community involvement in research phases is essential to uphold ethical standards and enhance trust and relevancy in findings [75]. However, the scope of Indigenous Data Sovereignty extends beyond research. It must also be applied to public health surveillance, health system planning, and digital infrastructures to ensure that data about Indigenous populations is governed in ways that align with Indigenous laws, protocols, and priorities aligned with specific Nations. Indigenous communities should have equitable access to their own data to support local decision-making, health planning, and service delivery. Embedding Indigenous data governance at community, institutional, and policy levels enables Indigenous-led responses to health challenges and advances broader goals of health equity and reconciliation [76]. If Indigenous data sovereignty is not incorporated into digital health education, there is a risk of exploiting or misusing Indigenous health data without proper consent or oversight, potentially undermining trust and perpetuating historical injustices in health care. Health care students’ understanding of Indigenous data sovereignty may be assessed through case studies or reflective essays where they analyze the importance of respecting Indigenous peoples’ control over their health data, explore ethical considerations, and demonstrate how to apply culturally appropriate practices in digital health initiatives (Table 1).

### Health Literacy and Patient Education

Digital health literacy is vital for patients, caregivers, and health care professionals alike. Patients need digital health literacy to manage their care effectively. Providers, leaders, and researchers must understand and use digital tools, as literacy across these groups supports quality care, informed decision-making, and research innovation. Digital health literacy fosters improved understanding, adherence, and outcomes across the health care system [50]. If health literacy and patient education are not incorporated into digital health education, patients may struggle to understand and effectively use digital health tools, exacerbating health disparities and hindering efforts to promote equitable access to care and tailor education to diverse literacy levels and needs. Health literacy and patient education in relation to digital health could be assessed through role-playing exercises or patient simulation scenarios where health care students demonstrate their ability to effectively communicate digital health information, assess patient comprehension, and tailor education to diverse literacy levels and needs (Table 1).

### Integrated Digital Health Competencies for the Quintuple Aim

Digital health competencies, such as interoperability, data literacy, and person-centered care principles, overlap across the 5 domains of the Quintuple Aim, enhancing both patient and provider experiences. These competencies promote equitable patient-centered care by improving access, streamlining care processes, and supporting population health efforts. A comprehensive approach to digital health education enables health care professionals to meet the diverse, interconnected demands of modern health care, supporting both individual and population-level outcomes. Failing to create a comprehensive approach to digital health education in relation to the Quintuple Aim could lead to fragmented health care delivery, where inefficiencies persist, patient experiences, health outcomes remain unequal, and costs rise, ultimately hindering the achievement of improved health for all.

## Standardized Yet Customizable Digital Health Education

Standardized informatics competency-based curricula, as described by organizations such as the Canadian Association of Schools of Nursing, the International Medical Informatics Association and the American Medical Informatics Association, provide a crucial foundation for health informatics education [20,21,77]. However, the health care landscape varies significantly across regions, influenced by factors like technological infrastructure, health care systems, cultural norms, and local health priorities. These differences require that standardized curricula also provide additional opportunities for curricula customization, versatility, and variety of delivery methods [78-80], bridging academic learning and real-world application, and aligning curricula with evolving health care demands [20,78].

To align with the Quintuple Aim framework, educational delivery strategies should intentionally match the competency emphasis to each aim. For instance, competencies that address the “Improving Population Health” aim should be emphasized in programs targeting public health professionals, while “Reducing Costs” and “Enhancing Efficiency” may be prioritized in executive and administrative education. This alignment ensures that the education of health care professionals is not only technically robust but also strategically positioned to support each domain of the Quintuple Aim.

The integration of case-based learning (CBL) with competency-based education potentially offers an effective pedagogical strategy, especially in digital health education, where learners need to master both theoretical concepts and practical skills in an ever-evolving field. CBL engages students with practical scenarios that simulate real-world challenges, encouraging critical thinking, collaborative problem-solving, and decision-making. In complex and rapidly evolving environments like health care, competency-based education can provide context-rich scenarios that mimic real-life challenges, while CBE ensures that students meet predefined learning outcomes essential for professional competence [78-81].

Achieving a balance between standardized and customized education could also involve adopting a modular approach. Core competencies can be standardized across programs, while additional modules can be tailored to meet local needs. For instance, health care educational programs can integrate standardized content on digital ethics and health data infrastructure but offer elective modules or microcredentials on specific areas such as local legislative and regulatory policies, electronic health records design, or virtual care workflows relevant to regional health care priorities [80]. Digital competencies are also not uniformly applicable across all health professions. Direct care providers, such as nurses, physicians, and allied health professionals, require competencies focused on clinical decision support, patient communication, and digital documentation. Health administrators need competencies focused on systems-level data analytics, resource management, and technology implementation. Public health practitioners, in contrast, may require competencies related to population surveillance, data ethics, and cross-sectoral data integration. These variations highlight the need for educational frameworks that are both discipline-sensitive and practice-informed, ensuring that digital health education is relevant to specific roles while maintaining shared foundational competencies.

Collaborations between academia and industry can ensure that curricula stay relevant. Industry insights help shape competencies that meet real-world demands, while partnerships provide students with practical experiences through internships and projects. This alignment fosters a skilled workforce ready to integrate digital health technologies effectively [34,40].

Together with maintaining strong academic and health care system partnerships, a hybrid approach, combining core standardization with customization through CBL and modular content delivery, potentially offers a sustainable model for developing digital health curricula that align with both global standards and local needs, and ensures health care professionals are prepared to navigate the complexities of digital health care, ultimately contributing to more effective, equitable, and patient-centered care delivery.

## Conclusion

The development of a standardized digital health curriculum that integrates both core competencies and context-specific customization is essential for equipping health care professionals to thrive in a rapidly evolving technological landscape. As health care systems increasingly rely on digital health technologies to enhance patient care and improve outcomes, health care professionals should at minimum be competent in skills such as data management, interoperability, digital literacy, and virtual care delivery. Standardization ensures consistency and fosters interprofessional collaboration, while context-specific case studies and modular customization enable education programs to address specific health care priorities, such as rural health care or emerging technologies. Achieving a balance between these approaches will ensure that digital health education remains relevant, responsive, and capable of meeting both global standards and local needs. Ultimately, a digitally competent workforce will be crucial to advancing health care delivery, supporting innovation, and achieving sustainable health outcomes in individuals, communities, and populations.

## Abbreviations

AI: artificial intelligence
CBL: Case-Based Learning
OCAP: Ownership, Control, Access, Possession
OECD: Organisation for Economic Co-operation and Development
WHO: World Health Organization
